# In Situ Synthesis of Bi_2_S_3_/BiFeO_3_ Nanoflower Hybrid Photocatalyst for Enhanced Photocatalytic Degradation of Organic Pollutants

**DOI:** 10.3390/molecules28248007

**Published:** 2023-12-08

**Authors:** Rentao Zhou, Xinman Tu, Peng Zheng, Li Zhang, Zhenxing Zeng

**Affiliations:** 1 College of Environment Science and Engineering, Guilin University of Technology, Guilin 541004, China; 2 Key Laboratory of Jiangxi Province for Persistent Pollutants Control and Resources Recycle, Nanchang Hangkong University, Nanchang 330063, China; 17770042612@163.com (P.Z.);; 3 College of Environmental Sciences, Sichuan Agricultural University, Chengdu 611130, China

**Keywords:** photocatalysis, advanced oxidation processes, organic degradation, heterojunction

## Abstract

Photocatalytic degradation of Malachite Green oxalate (MG) in a water body is of significant importance to our health protection, as it could cause various serious diseases. However the photocatalytic activity of most catalysts is still unsatisfactory, due to the poor reactive oxygen species production as a result of sluggish charge separation. Here, innovative nanoflower-shaped Bi_2_S_3_/BiFeO_3_ heterojunctions are prepared via a facile sol–gel method, exhibiting an enhanced reactive oxygen species generation, which leads to the excellent photocatalytic performance toward MG degradation. We verify that interfacing BiFeO_3_ with Bi_2_S_3_ could form a fine junction and offers a built-in field to speed up charge separation at the junction area; as a result, this shows much higher charge separation efficiency. By virtue of the aforementioned advantages, the as-prepared Bi_2_S_3_/BiFeO_3_ heterojunctions exhibit excellent photocatalytic performance toward MG degradation, where more than 99% of MG is removed within 2 h of photocatalysis. The innovative design of nanoflower-like Bi_2_S_3_/BiFeO_3_ heterojunctions may offer new viewpoints in designing highly efficient photocatalysts for environmentally related applications.

## 1. Introduction

In recent years, the world has been grappling with escalating energy crises and environmental pollution issues, particularly the pervasive presence of organic pollutants that significantly affect people’s daily lives. As the organic pollutants in water bodies are mostly highly toxic and may cause various serious diseases, effectively eliminating harmful organic pollutants from the environment has become a paramount concern for the scientific community. As a safe and sustainable way for organic pollutant removal, photocatalytic degradation of organics following a radical oxidative manner in recent years has been intensively studied. The key to achieving highly efficient organic pollutant removal from a water body lies in the development of a cost-effective photocatalyst with high reactive radical generation efficiency [1,2]. Unfortunately, most widely used photocatalysts primarily respond to UV–visible irradiation, limiting their practicality under visible light. Consequently, the quest for highly efficient and stable visible-light-driven photocatalysts has emerged as a hot topic in the field of photocatalysis.

Recently, BiFeO_3_ photocatalysts have garnered attention, due to their lower energy bandgap, excellent chemical stability at room temperature, and superior carrier transport properties. BiFeO_3_ holds promise for catalytic applications under visible light. Research has shown that BiFeO_3_, with a bandgap of approximately 2.2 eV [3,4], exhibits a favorable response to visible light and has been employed for the optical degradation of organic pollutants in water, including the degradation of organic dyes [5,6,7,8,9,10,11,12,13]. To further enhance the photocatalytic activity of BiFeO_3_, researchers have explored various modification techniques. Common approaches include element doping [14,15,16], which can boost visible-light-driven photocatalytic activity but often fails to effectively suppress the recombination of photogenerated electron–hole pairs, resulting in limited solar energy utilization. Some scholars have also experimented with noble metal deposition methods [17,18]; however, the high cost of precious metals makes these approaches less practical for real-world applications. Additionally, these modifications require further optimization to achieve better responsiveness to the visible light spectrum. As a result, researchers have turned to the incorporation of various materials into BiFeO_3_ to form composite heterojunction structures, such as CuO [19], TiO_2_ [20,21], and graphene oxide (GO) [22,23], among others. These heterojunction structures have been effective in enhancing visible light photocatalytic activity by mitigating electron–hole-pair recombination. However, these heterojunctions still lack optimal reducibility and oxidizability for photogenerated electron–hole pairs.

Hence, the search for a new semiconductor material to create heterojunctions with stronger reducibility and oxidizability is imperative. Additionally, since BiFeO_3_ nanoparticles possess a limited specific surface area, finding a substance capable of effectively increasing its surface area is crucial for enhancing photocatalytic reactions. Based on these considerations, our research aims to improve the reducibility and oxidizability of BiFeO_3_ through the formation of heterojunction structures with other materials, while simultaneously increasing its surface area. Achieving this under economical, simple, and mild reaction conditions has been a primary objective in our field. Our investigation revealed that Bi_2_S_3_, with its narrow band gap (Eg = 1.38–1.71 eV) [24] and the ability to adjust heterojunction morphology, is an ideal material for combining with BiFeO_3_. While previous studies have explored the potential of Bi_2_S_3_ as a visible-light-driven photocatalyst in conjunction with other photocatalysts, such as BiOCl [25,26], ZnO [27], TiO_2_ [28], CdS [29], WO_3_ [30], BiFeO_3_/α-Fe_2_O_3_ [31], La_x_Ba_1−x_Sr_y_Cd_1−y_O_3±δ_ [32], Bi_2_WO_6_ [33], BiVO_4_ [34], and Bi_2_O_3_ [35], no reports exist on the Bi_2_S_3_/BiFeO_3_ heterojunction photocatalysis.

In this study, we employed the in situ growth method to synthesize a novel Bi_2_S_3_/BiFeO_3_ nanoflower structure, demonstrating enhanced photocatalytic performance under visible light irradiation. We employed MG as a model contaminant to evaluate the material’s efficiency. Furthermore, we comprehensively investigated the structure, composition, morphology, and mechanism of this novel photocatalyst.

## 2. Results and Discussion

**Figure 1:**
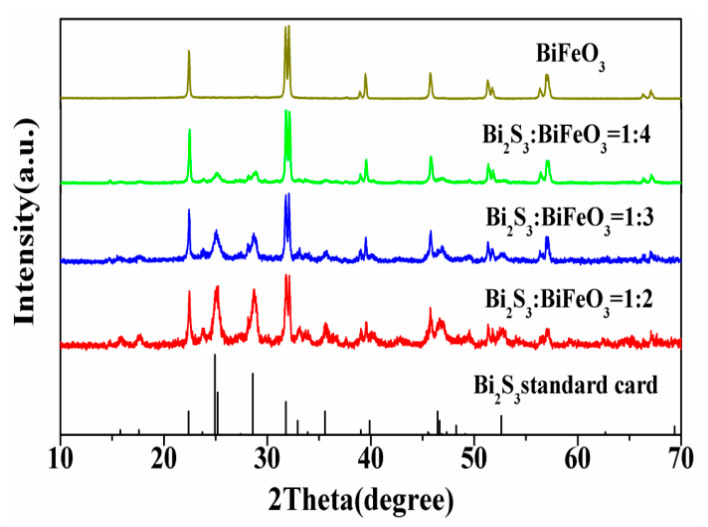
XRD patterns of Bi_2_S_3_/BiFeO_3_ with different proportions.

Figure 1 displays the X-ray diffraction (XRD) patterns of the Bi_2_S_3_/BiFeO_3_ samples with varying molar mass ratios, ranging from 1:2 to 1:4, in addition to pure BiFeO_3_, for comparison. The XRD patterns of pure BiFeO_3_ align well with the JCPDS 20-0169 standard [36], indicating the presence of characteristic diffraction peaks associated with this compound. In the composite material, discernible diffraction peaks attributed to Bi_2_S_3_ are evident, indicating the successful incorporation of Bi_2_S_3_ into the structure. It is worth noting that the diffraction peak of Bi_2_S_3_ at 2θ = 25.32 exhibits a slight overlap with BiFeO_3_ (2θ = 25.40). A noteworthy observation is that the positions of the Bi_2_S_3_ diffraction peaks at 25.2 and 28.6 display minor shifts towards larger angles in the composite material. These shifts suggest that Bi_2_S_3_ has effectively integrated with BiFeO_3_, leading to a reduction in the crystalline lattice spacing. Furthermore, it is important to acknowledge that the crystallinity of Bi_2_S_3_, synthesized via a hydrothermal method, is comparatively inferior to that of BiFeO_3_. These XRD findings shed light on the successful synthesis of Bi_2_S_3_/BiFeO_3_ composites and the structural modifications induced by their integration.

**Figure 2:**
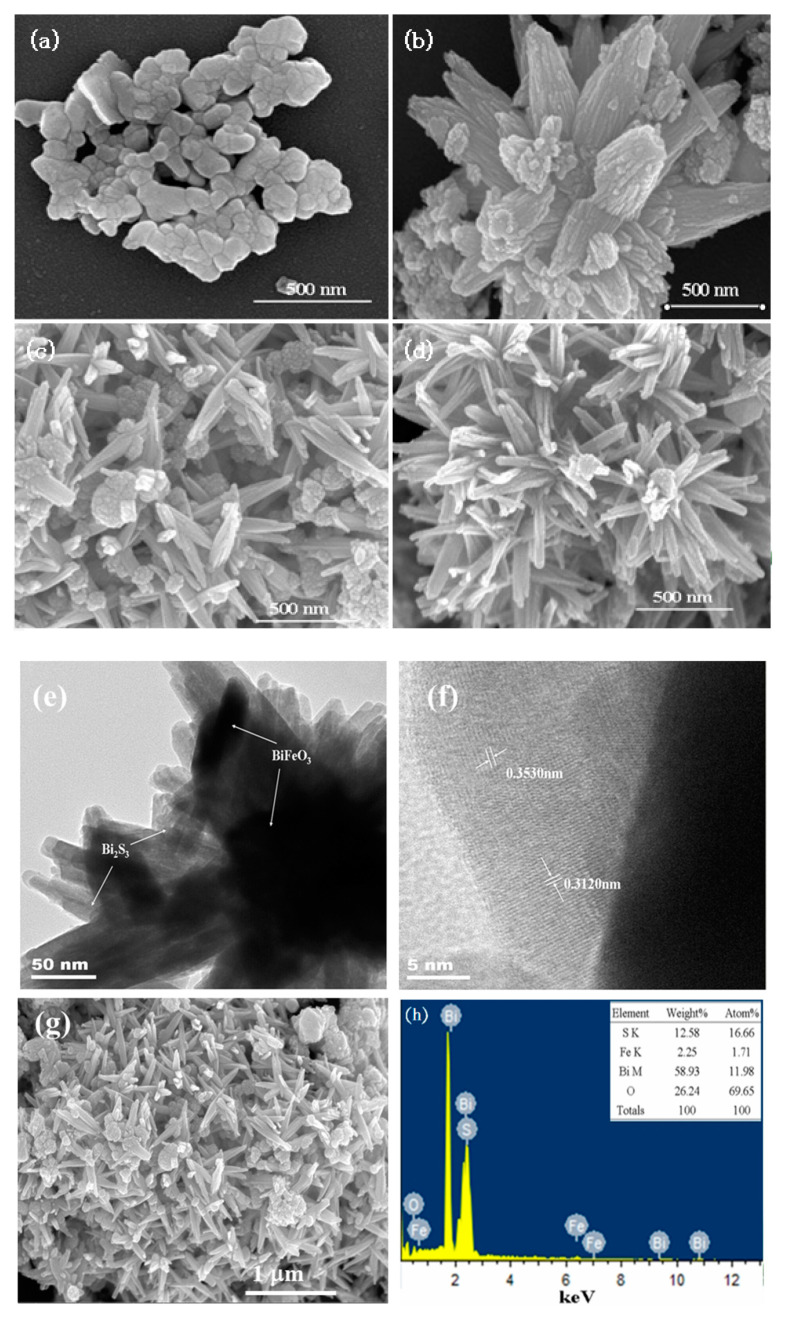
SEM images of samples (**a**) BiFeO_3_; (**b**) Bi_2_S_3_/BiFeO_3_(1:4); (**c**) Bi_2_S_3_/BiFeO_3_(1:3); (**d**) Bi_2_S_3_/BiFeO_3_(1:2); (**e**) TEM of Bi_2_S_3_/BiFeO_3_(1:3); (**f**) HRTEM of Bi_2_S_3_/BiFeO_3_(1:3); (**g**) the selected area of the EDX; (**h**) the EDX spectrum of the Bi_2_S_3_/BiFeO_3_(1:3).

The microcosmic morphologies and microstructure of both pure BiFeO_3_ and Bi_2_S_3_/BiFeO_3_ were meticulously examined using SEM, TEM, and HRTEM, revealing intriguing insights. SEM images of pure BiFeO_3_, as shown in Figure 2a, depict an assembly of particles characterized by smooth borderlines. However, the introduction of sulfur for the ion exchange process brought about significant changes in the morphology of the composite materials. As evidenced in Figure 2d, there is a noticeable shift towards a nearly spherical flower-like shape. Detailed TEM analysis, displayed in Figure 2e, unequivocally indicates the growth of Bi_2_S_3_ on the surface of BiFeO_3_ through ion exchange. HRTEM provided further elucidation, revealing that Bi_2_S_3_ exposes more planes corresponding to crystal planes (310) and (211), which correspond to lattice spacings of 0.3530 nm and 0.3120 nm, respectively. The formation mechanism of these changes can be understood as follows: Initially, the lattice of BiFeO_3_ is pristine, with filled bonding orbitals and relatively few dangling bonds at the interface, rendering the entire BiFeO_3_ molecule in a stable state. The introduction of sulfuric acid provides the necessary energy for the surface activation of BiFeO_3_. This activation leads to damage to the original crystal lattice, exposing elemental bismuth and creating an abundance of dangling bonds on the surface of BiFeO_3_. Consequently, surface energy increases significantly, enhancing surface activity. When L-cysteine is introduced, a critical chemical transformation occurs. The elemental sulfur in L-cysteine forms stable new chemical bonds with the elemental bismuth on the surface of BiFeO_3_, resulting in the formation of Bi_2_S_3_/BiFeO_3_ heterojunctions. Moreover, the EDX spectrum analysis corroborates these findings, as it reveals the presence of Bi, O, Fe, and S elements, further confirming the formation mechanism of the Bi_2_S_3_/BiFeO_3_ heterojunction.

**Figure 3:**
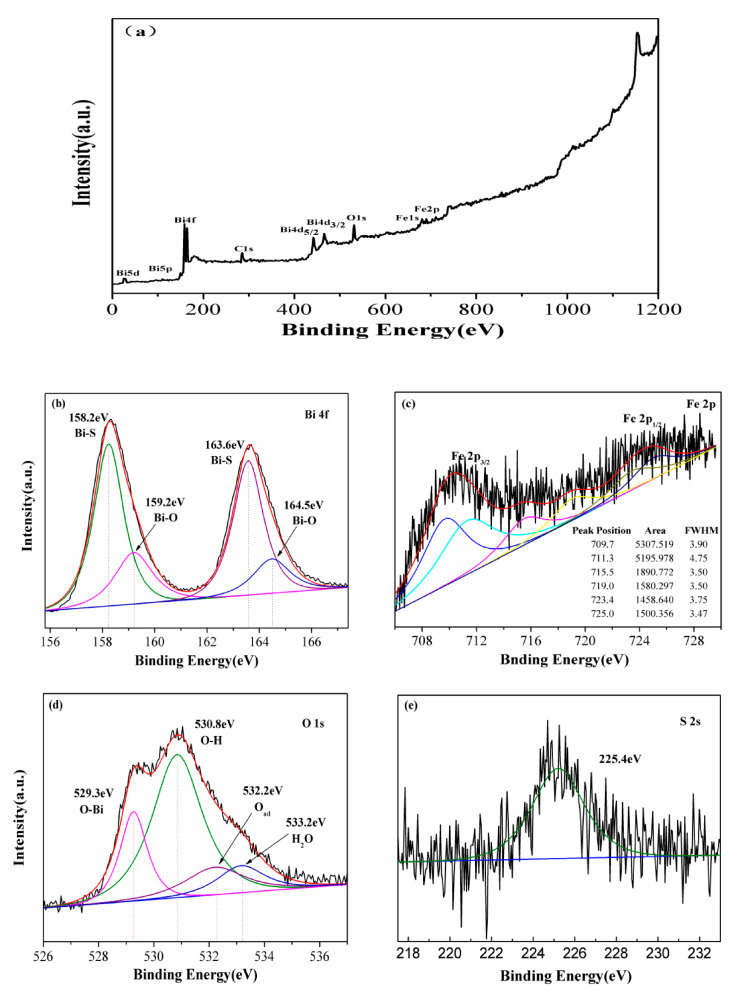
(**a**) Whole XPS spectra; (**b**) Bi(4f); (**c**) Fe(2p); (**d**) O(1s) and (**e**) S(2s) lines of the Bi_2_S_3_/BiFeO_3_.

To gain further insight into the surface composition of the heterojunction material, X-ray photoelectron spectroscopy (XPS) was employed, providing compelling evidence for the coexistence of Bi_2_S_3_ and BiFeO_3_, as well as the presence of Bi, Fe, O, and S elements (Figure 3). The high-resolution XPS spectra reveal overlapping peaks centered at 158.2 eV and 163.6 eV, as well as 159.2 eV and 164.5 eV. These peaks can be attributed to the binding energy of Bi^3+^ 4f_7/2_ and 4f_5/2_ in Bi_2_S_3_ [28] and the Bi-O bond in BiFeO_3_ [37,38], respectively. The XPS spectrum of Fe(2p) is noteworthy, exhibiting six peaks at 709.9, 711.3, 715.5, 719.0, 723.4, and 725.0 eV. Notably, the Fe(2p) peaks at binding energies of 711.3/725.0 eV and 709.9/723.4 eV, along with a satellite signal peak at 719.0 eV and 715.5 eV, are characteristic of Fe^3+^ and Fe^2+^. The Fe(2p) spectra primarily feature two maxima peaks corresponding to the electron levels of Fe 2p_3/2_ and Fe 2p_1/2_ [39,40]. It is worth noting that the peak of the high-spin Fe^3+^ ion is significantly broader, compared to the low-spin Fe^2+^ ions. This broadening can be attributed to electrostatic interactions and spin–orbit coupling among the 2p empty orbital and the unpaired 3d electrons of iron ions. In the XPS spectrum of O 1s, four distinct peaks were observed at 529.3, 530.8, 532.2, and 533.2 eV, corresponding to oxygen–metal bonds in the lattice O_2_^−^ of BiFeO_3_ [41], remaining OH^−^ at the material surface [42], adsorbed oxygen (O_ads_), and surface-adsorbed H_2_O [43]. The presence of a smaller-intensity peak at a binding energy of 225.4 eV in the S 2s spectrum can be attributed to the S-Bi bond of Bi_2_S_3_ [44]. In conclusion, the XPS analysis unequivocally confirms the successful growth of Bi_2_S_3_ nanomaterials on the surface of BiFeO_3_, forming the Bi_2_S_3_/BiFeO_3_ heterojunction.

**Figure 4:**
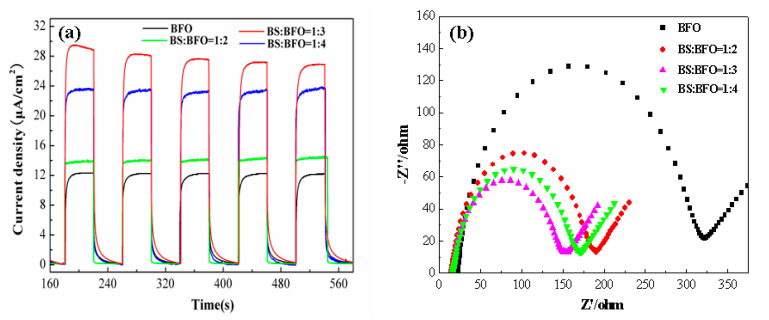
Photocurrents (**a**) BiFeO_3_ and the proportions of Bi_2_S_3_ and BiFeO_3_ ([Na_2_SO_4_] = 0.5 M) and (**b**) electrochemical impedance spectroscopy (EIS) for BiFeO_3_ and the proportions of Bi_2_S_3_ and BiFeO_3_ ([Na_2_SO_4_] = 0.5 M, [K_4_Fe(CN)_6_•3H_2_O] = 2.5 mM, [K_3_[Fe(CN)_6_]] = 2.5 mM).

Photocurrent measurements were conducted for both pure BiFeO_3_ and various molar ratios of Bi_2_S_3_/BiFeO_3_, to probe electronic interactions, as depicted in Figure 4a. The composite materials exhibited markedly higher photocurrent responses compared to single BiFeO_3_ during on–off cycles under visible-light irradiation. This enhancement can be directly attributed to the improved separation efficiency of photo-generated electrons within the heterojunction. Notably, when the molar mass ratio of Bi_2_S_3_/BiFeO_3_ is 1:3, the photocurrent response surpassed that of the other ratios, signifying superior performance in charge separation. This enhanced photocurrent in the Bi_2_S_3_/BiFeO_3_ heterojunction indicates heightened efficiency in separating electron–hole pairs, thereby enhancing the photocatalytic properties. To delve further into the charge separation process, electrochemical impedance spectroscopy (EIS) was employed. In EIS, a smaller arc radius signifies more effective separation of photo-generated electron–hole pairs and faster interface charge transfer.

Figure 4b illustrates the fact that the arc radius of the Bi_2_S_3_/BiFeO_3_ heterojunction is notably smaller than that of single BiFeO_3_. This difference is particularly pronounced when the ratio of Bi_2_S_3_/BiFeO_3_ is 1:3, where the arc radius is minimized. These results underscore the significant role played by the Bi_2_S_3_/BiFeO_3_ heterojunction in improving the separation of photogenerated electron–hole pairs and facilitating efficient charge transfer at the interface. Consequently, the utilization of light energy is greatly enhanced, contributing to the overall photocatalytic efficiency.

**Figure 5:**
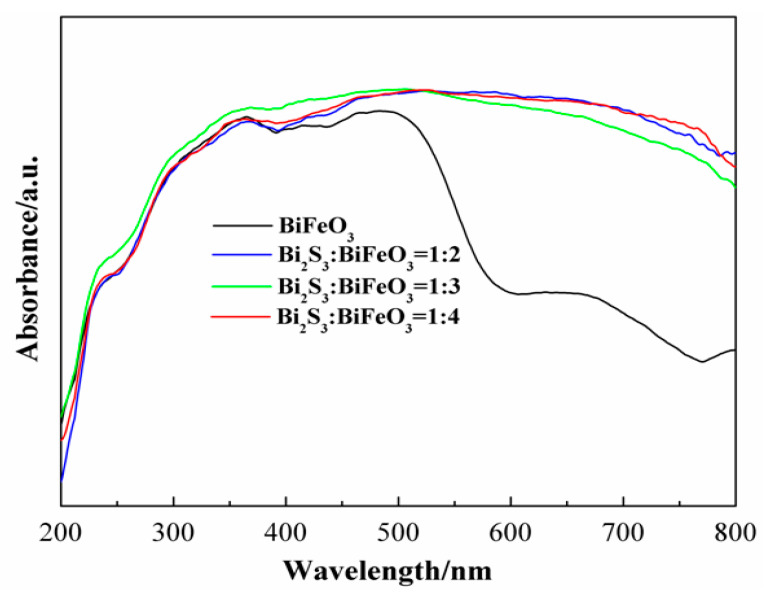
UV-vis diffuse reflection spectra of pure BiFeO_3_ and the different proportions of Bi_2_S_3_/BiFeO_3_.

The UV-Vis DRS spectra of the Bi_2_S_3_/BiFeO_3_ heterojunction photocatalysts, with varying proportions, along with pure BiFeO_3_, are presented in Figure 5. These spectra offer valuable insights into the optical properties of the materials. The optical absorption spectra, as revealed by DRS, demonstrate distinct characteristics. The pure BiFeO_3_ sample exhibits intrinsic semiconductor-like absorption, primarily in the orange region of the visible spectra. In contrast, the Bi_2_S_3_/BiFeO_3_ heterojunction composite materials exhibit absorption across the entire visible region of the spectrum, indicating enhanced light absorption capabilities. Notably, pure BiFeO_3_ shows an absorption edge at approximately 610 nm, corresponding to a band gap of 2.03 eV for BiFeO_3_. In the case of the Bi_2_S_3_/BiFeO_3_ heterojunction, the absorption edge shifts towards the visible light region with increased intensity, compared to BiFeO_3_. This shift signifies the photosensitization effect of Bi_2_S_3_ on BiFeO_3_, where Bi_2_S_3_ enhances the absorption of visible light by the composite material. These findings emphasize the significant role of Bi_2_S_3_ in expanding the spectral range of light absorption by the BiFeO_3_ photocatalyst, thereby enhancing its photocatalytic potential.

**Figure 6:**
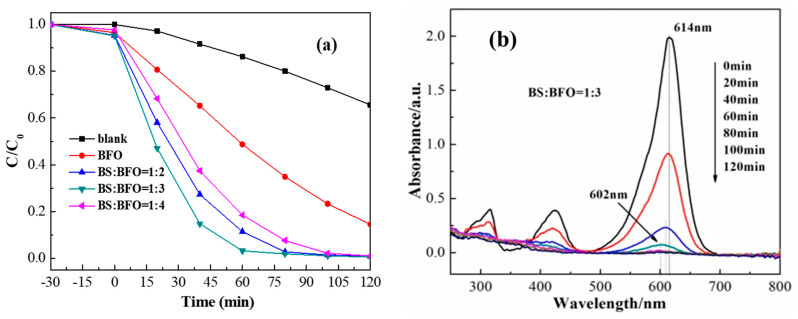
(**a**) Photocatalytic degradation of MG under visible-light irradiation using Bi_2_S_3_/BiFeO_3_ heterojunctions as the catalyst; (**b**) the full-wavelength scanning of Bi_2_S_3_/BiFeO_3_(1:3) heterojunction photocatalyst.

The photocatalytic activity of the Bi_2_S_3_/BiFeO_3_ heterojunction with varying proportions of Bi_2_S_3_ was investigated through the photodegradation of the organic pollutant MG, as illustrated in Figure 6a. In the absence of the catalyst, the rate of photodegradation of MG under visible light (λ > 420 nm) irradiation remained below 40% within 2 h. However, when the Bi_2_S_3_/BiFeO_3_ heterojunction catalysts were introduced, notable improvements were observed. MG was completely photodegraded after 60 min, demonstrating the enhanced photocatalytic efficiency of these materials. The photocatalytic performance followed the following order: Bi_2_S_3_/BiFeO_3_ (1:3) > Bi_2_S_3_/BiFeO_3_ (1:2) > Bi_2_S_3_/BiFeO_3_ (1:4) > BiFeO_3_. In particular, Bi_2_S_3_/BiFeO_3_ (1:3) exhibited the highest photocatalytic efficiency, with approximately 99% of MG molecules decomposed within 60 min under visible light. This result underscores the significance of the optimal proportion between BiFeO_3_ and Bi_2_S_3_ for enhanced photocatalytic activity. Scanning electron microscopy revealed that Bi_2_S_3_/BiFeO_3_ (1:3) possessed a more uniform morphology, further supporting the superior photocatalytic performance observed. Moreover, full-wavelength scanning in Figure 6b highlights the degradation process: as MG was degraded by the addition of the Bi_2_S_3_/BiFeO_3_ photocatalyst, the UV–visible absorption peak gradually weakened until it disappeared. During this weakening, there was a noticeable blue shift in the UV–visible absorption peak, indicating the generation of intermediate products in the optical degradation process, which, however, did not impede the degradation process. These findings underscore the substantial photocatalytic advantage of the Bi_2_S_3_/BiFeO_3_ heterojunction, particularly when the proportion is optimized, and the importance of uniform particle surfaces in enhancing photocatalytic activity.

**Figure 7:**
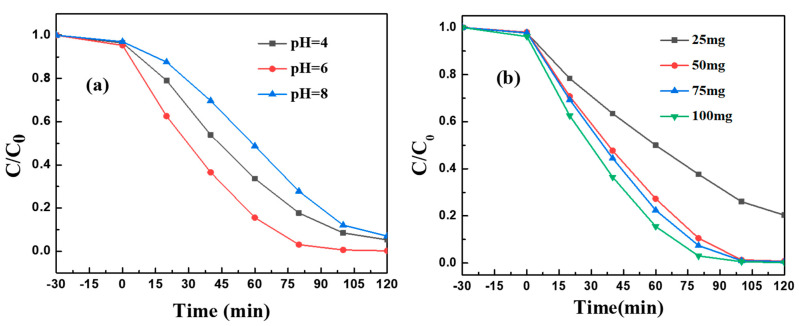
Photocatalytic performance of Bi_2_S_3_/BiFeO (1:3) toward MG degradation at (**a**) various pH conditions. (**b**) Effects of catalyst dosage on MG degradation.

As shown in Figure 7a, Bi_2_S_3_/BiFeO (1:3) shows pH-dependent photocatalytic activity toward MG degradation, where the heterojunction catalyst exhibits excellent catalytic activity, with a pH around 6. An acidic or alkaline condition brings a negative effect toward MG degradation, which may be attributed to the fact that the heterojunction catalyst is not stable enough against acid or alkaline corrosion. We also investigate the influence of catalyst dosage on the MG photo degradation, and the results are shown in Figure 7b. It is easy to find that, with the increase in the amount of catalyst used, the catalytic activity of heterojunctions toward MG degradation increases, which may be attributed to the increased catalyst amount, which could provide a more active side for oxygen activation to generate more reactive oxygen species. It should be noted here that an excess amount of catalyst would bring a light shading effect, which may bring negative effects toward pollutant removal. As can be seen from Figure 7b, limited activity improvement is found with the amount of catalyst increasing from 75 mg to 100 mg, and we believe that further increasing the amount of catalyst would not bring much improvement in catalytic activity. Thus, we then chose 100 mg of catalyst as the optimized condition to carry out other experiments.

**Figure 8:**
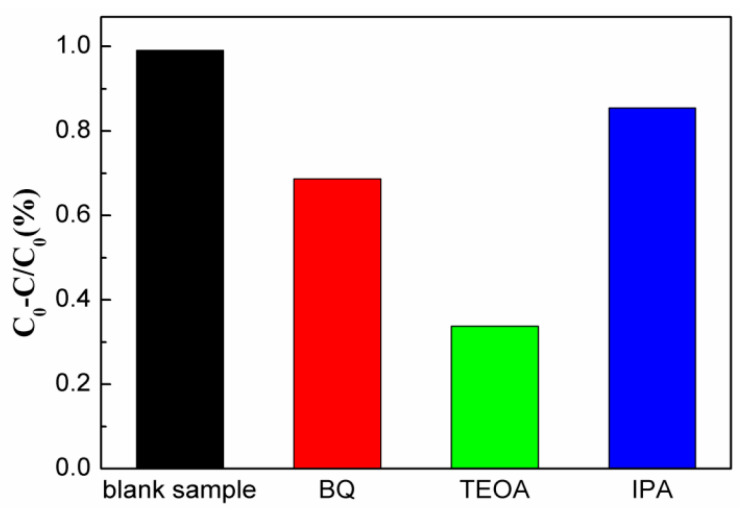
Effects of various scavengers on the photocatalytic efficiencies of Bi_2_S_3_/BiFeO_3_ in MG degradation under visible-light irradiation.

To gain a deeper understanding of the photodegradation mechanism, various sacrificial agents were introduced to elucidate the roles of specific reactive groups in the reaction process. As depicted in Figure 8, when isopropanol (IPA) was introduced as a sacrificial agent to capture hydroxyl (•OH) radicals, the degradation of MG was almost negligible. This observation suggests that •OH radicals play a crucial role in the degradation process, as they are potent oxidants, capable of effectively oxidizing many organic pollutants. Conversely, when triethanolamine (TEOA) was added to capture the photo-generated holes (h^+^), and 4-benzoquinone (BQ) was used to capture •O_2_^−^ radicals, the efficiency of MG degradation was significantly reduced. This reduction was particularly pronounced in the presence of TEOA. These results imply that, during the photodegradation reaction, •O_2_^−^ radicals and photo-generated holes (h^+^) work in tandem to facilitate the degradation of organic pollutants. The •OH radical, being a robust oxidant capable of oxidizing a wide range of organic pollutants, is likely a key contributor to the degradation process. These findings align with the existing literature, further supporting the significance of •OH radicals in the photodegradation of organic pollutants.

**Figure 9:**
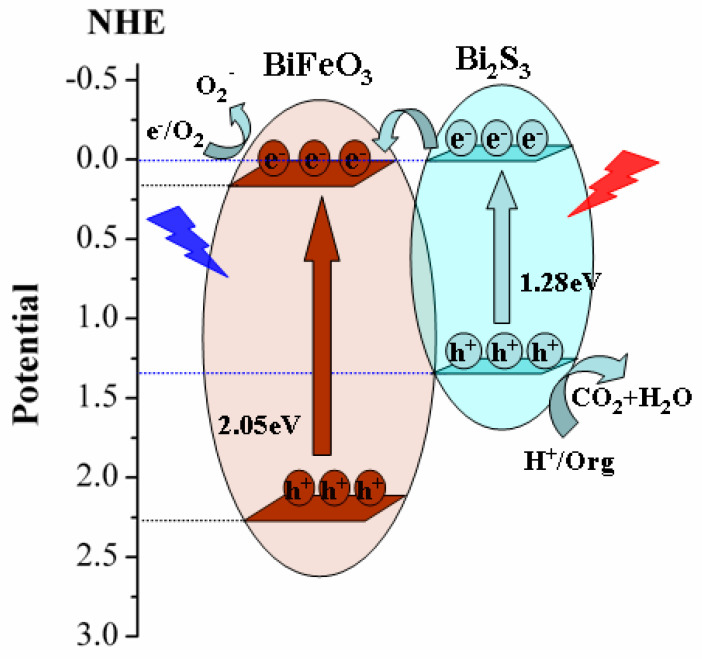
Schematic illustration of the Bi_2_S_3_/BiFeO_3_ photocatalytic reaction process under visible-light irradiation.

To provide further authentication of the photocatalytic mechanisms at play, several key energy levels and oxidation–reduction potentials were considered (Figure 9). The band gap of BiFeO_3_ was determined to be 2.05 eV, based on UV-Vis DRS spectra. Additionally, the conduction band of BiFeO_3_ is at +0.21eV (E_CB_ = +0.21 eV) [45], and the valence band is at +2.26 eV (E_VB_ = +2.26 eV). In contrast, Bi_2_S_3_ has a conduction band at 0 V (E_CB_ = 0 V) [46] and a valence band at +1.38 eV (E_VB_ = +1.38 eV) [47]. Notably, the conduction band of Bi_2_S_3_ is more negative than that of BiFeO_3_. Based on the above energy-band structure analysis, the photogenerated electrons are transferred from the conduction band of Bi_2_S_3_ to the conduction band of BiFeO_3_, due to the more negative conduction band of Bi_2_S_3_. This facilitates the generation of •O_2_^−^ on the material surface by oxygen absorption, which can then directly oxidize the target contaminant MG. On the other hand, the oxidation–reduction potential of •OH is 2.38 V [48], significantly more positive than the valence band of BiFeO_3_ (E_VB_ = +2.26 V) [49] and Bi_2_S_3_ (E_VB_ = +1.38 eV) [50]. Consequently, the photoexcited Bi_2_S_3_/BiFeO_3_ generates holes that are unable to oxidize water to form hydroxyl radicals. Furthermore, the valence band energy of Bi_2_S_3_ is lower than that of BiFeO_3_. Thus, the photogenerated holes in the valence band of BiFeO_3_ are transferred to the valence band of Bi_2_S_3_. This energy transfer enables the direct oxidation of MG. Considering the properties of each substance in the reaction system, these insights suggest the procedures and mechanisms of the reaction [50].

(1) $$Bi_{2}S_{3}+hv\to h_{Bi_{2}S_{3}}^{+}+e_{Bi_{2}S_{3}}^{-}$$

(2) $$BiFeO_{3}+hv\to h_{BiFeO_{3}}^{+}+e_{BiFeO_{3}}^{-}$$

(3) $$e_{Bi_{2}S_{3}}^{-}+BiFeO_{3}\to e_{BiFeO_{3}}^{-}$$

(4) $$e_{BiFeO_{3}}^{-}+O_{2}\to •O_{2}^{-}$$

(5) $$•O_{2}^{-}+MG\to products$$

(6) $$h_{BiFeO_{3}}^{+}+Bi_{2}S_{3}\to h_{Bi_{2}S_{3}}^{+}$$

(7) $$h_{Bi_{2}S_{3}}^{+}+MG\to products$$

## 3. Experimental

### 3.1. Material Synthesis

BiFeO_3_ was prepared according to the reported method, with some modification [51]. Typically, 5 mM of bismuth nitrate pentahydrate (Bi(NO_3_)_3_•5H_2_O) and 5 mM of ferric nitrate (Fe(NO_3_)_3_•9H_2_O) were added into 35 mL of ethylene glycol monomethyl ether, to form a fine mixture. The solution was vigorously stirred until it reached a state of homogeneity, resulting in a transparent and stable solution at room temperature. Subsequently, the sample was left to dry for 24 h at 80 °C, yielding a gel-like powder. This powder was then carefully ground and subjected to heating in a muffle furnace at 550 °C for 4 h. After the heating process, the sample was allowed to cool to room temperature, and its final form was obtained through additional grinding.

**Figure 10:**
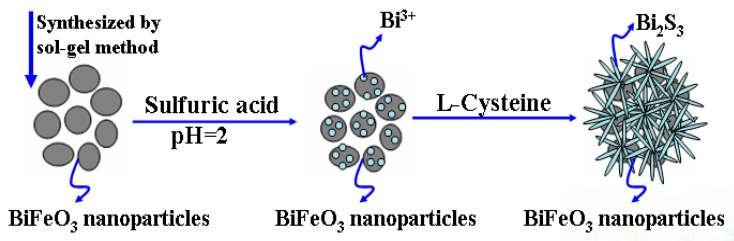
Schematic illustration of the Bi_2_S_3_/BiFeO_3_ synthetic processes.

Following this initial preparation, the next step involved the synthesis of Bi_2_S_3_/BiFeO_3_ through an in situ growth process on the BiFeO_3_ substrate under hydrothermal conditions, as depicted in Figure 10. 

Commencing with three clean beakers, each received 35 mL of deionized water. Sequentially, L-cysteine was introduced at concentrations of 6 mM, 4 mM, and 3 mM into the respective beakers. Complete dissolution of L-cysteine was ensured. Sulfuric acid was employed to carefully adjust the pH level to 2, while maintaining precision in this process. With meticulous care, an equivalent quantity of BiFeO_3_ (4 mM) was gradually added to each solution. Stirring continued until the BiFeO_3_ was uniformly dispersed within the mix. This stirring process endured for an additional 30 min. The mixed solutions were then carefully transferred to a 50 mL autoclave. The autoclave was subjected to a hydrothermal reaction at 150 °C for a duration of 12 h. Following completion of the reaction, the contents underwent filtration, and the resulting material was thoroughly washed with deionized water and absolute ethanol. The Bi_2_S_3_/BiFeO_3_ heterojunction materials obtained from the process were dried at 80 °C for 12 h under ambient air conditions. The final samples were categorized as Bi_2_S_3_/BiFeO_3_ (1:2), Bi_2_S_3_/BiFeO_3_ (1:3), and Bi_2_S_3_/BiFeO_3_ (1:4), respectively, based on the ratios used during their preparation.

### 3.2. Characterization

The crystalline phase of the samples was investigated using X-ray diffraction (XRD, D8 Advance, Bruker, Mannheim, Germany), using graphite monochromatized Cu-Kα (λ = 1.5406 Å) radiation at 40 kv and 40 mA with a scan rate of 2 °/min between 10° and 70°. The UV–vis diffuse reflectance spectra (DRS) of the powders were collected using a U-3900/3900H UV-vis spectrophotometer (UV-vis DRS, U-3900/3900H, Hitachi High-Technologies, Tokyo, Japan) equipped with an integrating sphere attachment. The general morphologies and micro-area chemical analysis of the samples were obtained using scanning electron microscopy images (SEM, Sirion 200, FEI, Eindhoven, The Netherlands) and energy-dispersive X-ray spectroscopy (EDS) with a scanning voltage of 5.00 kV. Further microstructure and crystallinity of the sample was analyzed using transmission electron microscopy (TEM, Tecnai F20, FEI, Hillsboro, OR, USA) and high-resolution transmission electron microscopy (HRTEM) observation. The chemical states of as-prepared photocatalysts were tested using monochromated Al-Kα X-ray photoelectron spectroscopy (XPS, Axis Ultra DLD) at a residual gas pressure of less than 10^−8^ Pa.

Electrochemical measurements were performed on a CHI 660D electrochemical workstation (Shanghai Chenhua, Shanghai, China) by using a standard three-electrode system with a working electrode. (The working electrode was prepared by dip-coating on a 3 cm × 1 cm fluorine tin oxide (FTO) glass electrode, with the films drying under room condition.) A graphite electrode was used as the electrodes, and a standard calomel electrode in saturated KCl was used as a reference electrode. A total of 0.5 M sodium sulfate was used as the electrolyte solution for the photocurrent test, and 0.5 M sodium sulfate, 2.5 mM of potassium ferricyanide and potassium ferrocyanide were adopted as the supporting electrolyte for Ac impedance.

### 3.3. Photocatalytic Activity Evaluation

The effectiveness of the Bi_2_S_3_/BiFeO_3_ heterojunction catalysts in photocatalysis was assessed through the degradation of Malachite Green oxalate (MG) under visible light conditions (λ > 420 nm). These photocatalytic reactions were conducted using simulated solar light with an intensity equivalent to 300 W, generated by a Xe-lamp system from Asahi Spectra Co., Ltd. (Torrance, CA, USA) The initial concentration of the Malachite Green solution was set at 20 mg/L. The photocatalytic procedure was performed in a homemade reactor with catalytic conditions quite similar to the reported method; typically, 0.1 g of the catalyst was dispersed into 100 mL of the Malachite Green oxalate (MG) solution [52]. To establish a balance between the adsorption and desorption of Malachite Green on the catalyst surface, the mixture was magnetically stirred for 30 min in the dark.

At specified irradiation time intervals, approximately 5 mL of the suspension was extracted every 10 min. These samples were subsequently centrifuged to obtain clear solutions, which were then analyzed using a UV–vis spectrophotometer (UV-vis DRS, U-3900/3900H, Hitachi High-Technologies, Tokyo, Japan). The maximum absorbance of the solution was determined at 614 nm. To provide a basis for comparison, parallel experiments using pure BiFeO_3_ were conducted alongside the Bi_2_S_3_/BiFeO_3_ experiments, allowing for an evaluation of the relative performance of Bi_2_S_3_/BiFeO_3_, compared to pure BiFeO_3_.

## 4. Conclusions

In summary, we have shown the first instance of fabricating nanoflower-shaped Bi_2_S_3_/BiFeO_3_ heterojunctions via a simple sol–gel process. The formed type II heterojunction is found capable of providing a powerful built-in field to accelerate electron–hole spatial separation, which leads to the enhanced reactive oxygen species generation. As a result, the as-prepared Bi_2_S_3_/BiFeO_3_ heterojunctions exhibit excellent photocatalytic performance toward MG degradation, where more than 99% of MG is removed within 2 h of photocatalysis. The innovative design shown in this work may offer new viewpoints in designing highly efficient photocatalysts for environmental purification. For real water purification, however, we still need to immobilize the catalyst, so as to prevent catalyst loss. The immobilization of the as-prepared Bi_2_S_3_/BiFeO_3_ on a light-weight carrier, for example loofah, to construct floating materials, is another possibility for its practical application.

## Data Availability

Data are contained within the article.
